# Evaluation of magnetic nanoparticle samples made from biocompatible ferucarbotran by time-correlation magnetic particle imaging reconstruction method

**DOI:** 10.1186/1471-2342-13-15

**Published:** 2013-06-05

**Authors:** Yasutoshi Ishihara, Takumi Honma, Satoshi Nohara, Yoshio Ito

**Affiliations:** 1 School of Science and Technology, Meiji University, Higashimita Tama, Kawasaki, Kanagawa, Japan; 2 Graduate School of Science and Technology, Meiji University, Higashimita, Tama, Kawasaki, Kanagawa, Japan; 3 The Nagoya Research Laboratory, Meito Sangyo Co., Ltd, Kaechi Nishibiwajima, Kiyosu, Aichi, Japan

**Keywords:** Magnetic particle imaging, MPI, Nanoparticle, Ferucarbotran, Resovist, Image reconstruction, Time correlation

## Abstract

**Background:**

Molecular imaging using magnetic nanoparticles (MNPs)—magnetic particle imaging (MPI)—has attracted interest for the early diagnosis of cancer and cardiovascular disease. However, because a steep local magnetic field distribution is required to obtain a defined image, sophisticated hardware is required. Therefore, it is desirable to realize excellent image quality even with low-performance hardware. In this study, the spatial resolution of MPI was evaluated using an image reconstruction method based on the correlation information of the magnetization signal in a time domain and by applying MNP samples made from biocompatible ferucarbotran that have adjusted particle diameters.

**Methods:**

The magnetization characteristics and particle diameters of four types of MNP samples made from ferucarbotran were evaluated. A numerical analysis based on our proposed method that calculates the image intensity from correlation information between the magnetization signal generated from MNPs and the system function was attempted, and the obtained image quality was compared with that using the prototype in terms of image resolution and image artifacts.

**Results:**

MNP samples obtained by adjusting ferucarbotran showed superior properties to conventional ferucarbotran samples, and numerical analysis showed that the same image quality could be obtained using a gradient magnetic field generator with 0.6 times the performance. However, because image blurring was included theoretically by the proposed method, an algorithm will be required to improve performance.

**Conclusions:**

MNP samples obtained by adjusting ferucarbotran showed magnetizing properties superior to conventional ferucarbotran samples, and by using such samples, comparable image quality (spatial resolution) could be obtained with a lower gradient magnetic field intensity.

## Background

Developments in nanotechnology have been exploited to realize innovative techniques for the diagnosis and treatment of diseases in the field of medicine. In particular, nanotechnology has been applied to drug delivery systems (DDSs) in which a nanoparticle, the surface of which is functionalized with various antibodies, is used to attack cancer cells; furthermore, cellular imaging using the light scattered by a nanoparticle has been actively studied [1]. Cancer treatment has also been attempted using nanoparticles with high sensitivity to light or heat [2,3]. Similarly, the use of magnetic nanoparticles (MNPs) has also been investigated. For example, in the thermal treatment of cancer, MNPs are used as heating elements to selectively heat a cancer cell [4]; in fact, clinical trials of this technique are now underway [5]. Gleich *et al.* reported magnetic particle imaging (MPI), a technique in which MNPs are applied to medical imaging [6,7]. MPI uses the harmonic components of the magnetization signal produced by the interaction between the nonlinear magnetizing properties of an MNP and the alternative magnetic field around the target body. In this technique, MNPs play the role of a contrast medium in blood vessels for the diagnosis of cardiovascular diseases and that of a tracer that images the distribution of MNPs accumulated in the cancer cell. Owing to its various advantages, MPI has attracted considerable research attention as a new diagnostic imaging modality.

The possibility of *in-vivo* real time imaging has already been demonstrated in a mouse [8]. However, a clinical MPI system for humans will require a large magnetic field generator to realize a magnetic field distribution with a steep slope, which is advantageous in identifying the position of an MNP and in obtaining a high-resolution image in MPI. To avoid this problem, the segmentation scanning of the objective region has been proposed as a workaround [9].

In order to realize a feasible clinical system, since 2007, we have focused our attention on developing a high-resolution MPI imaging system that does not require special, high-performance hardware. As a candidate procedure, we have proposed an image reconstruction method to improve the spatial resolution by reducing the interference signal produced around the target region [10]. Through the use of this method, local image artifacts and blurring could be suppressed. Moreover, we reported that the components of image blurring and artifacts could be suppressed based on the difference of the “saturation time” between the ideal magnetization signal (corresponding to an impulse response or a point spread function (PSF) of the MPI system), which arises from an isolated MNP and the observed magnetization signal [11]. However, when MNPs are distributed continuously, it becomes difficult to obtain an accurate image of MNPs because of the enhancement of the image edge part, as noted previously. Therefore, we have proposed a new image reconstruction method and evaluated its validity [12]. In this method, the observed magnetization signal produced around a target region is extracted based on the correlation with a system function, and it can be reflected by the intensity of the reconstruction image. However, because the image reconstruction is performed based on a simple correlation, it tends to expand the image blurring theoretically. Therefore, it is necessary to remove the image blurring actively, and we are currently attempting to design an effective algorithm for this purpose [13].

Meanwhile, to improve the image resolution without requiring high-performance hardware, the characteristics of an MNP should be improved in parallel to the image quality improvement by such an image reconstruction method because the spatial broadening of the observed magnetization signal is approximated by the differentiation of the Langevin function [14]. Therefore, high spatial resolution is expected when the particle diameter of an MNP is large because the full width at half maximum (FWHM) of this differentiated waveform narrows with an increase in the particle diameter [15].

Currently, the ferucarbotran (a drug substances of Resovist; supplied only by Meito Sangyo Co., Ltd.) used as a contrast medium for magnetic resonance imaging (MRI) is being used in MPI. However, because the particle diameters of the MNPs contained in ferucarbotran differ, as already pointed out, it is not an optimal contrast medium for demonstrating the performance of MPI. Generally, if the influence of the relaxation time for the magnetization response is ignored, the magnetization properties of an MNP with large particle diameter are advantageous for MPI [16]. Therefore, a trial in which MNPs with large particle diameters are compounded efficiently using an organic solvent is performed [17]. However, sufficient information regarding the biocompatibility of most particles compounded by such processes is not available, and it is expected that obtaining such information will require considerable effort and time. On the other hand, some studies have shown that the signal detection sensitivity in magnetic particle spectroscopy (MPS) can be enhanced by using fractionation samples of ferucarbotran [18] or FeraSpin (Miltenyi Biotec GmbH) [19].

In this study, MNP samples adjusted to some particle diameters are prepared by using ferucarbotran, which has already been approved for clinical use, as a base material. In particular, this study aims at estimating the influence of the characteristics of the MNPs based on the difference in particle diameter on the images reconstructed using our proposed method in the numerical simulation and the experiments using a prototype. In addition, the relation between the characteristics of the MNP and the hardware ability is discussed based on the results of such reconstructed images.

### Principle

#### Time-correlation MPI reconstruction method

**Figure 1:**
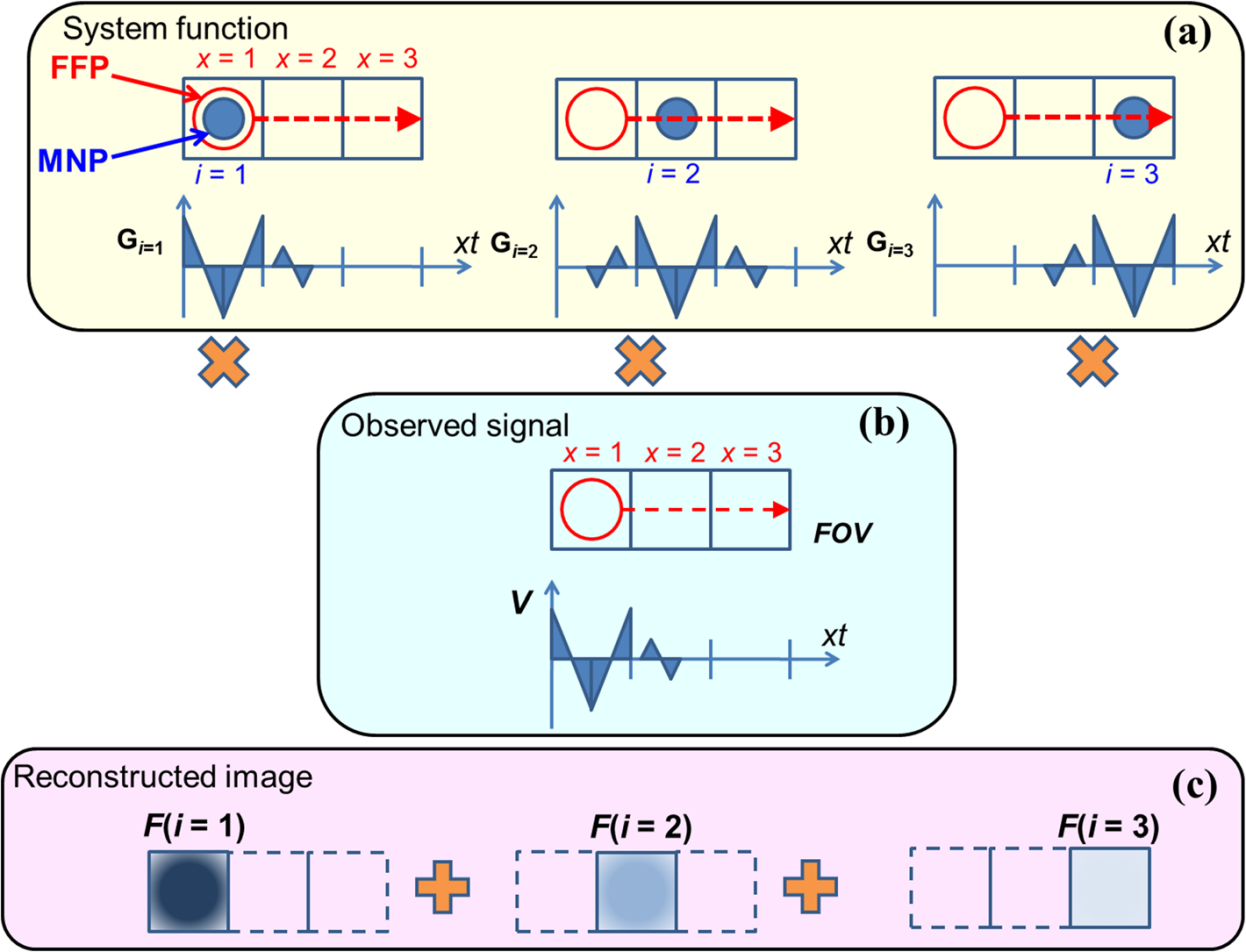
**Concept of image reconstruction by time-correlation method.** (**a**) The waveforms of the induced electromotive force produced by the MNPs arranged at each point are observed by scanning the FFP, and the signal sequences that connect them to the time axis are defined as the system function. (**b**) The signal detected from an unknown MNP distribution at each FFP is connected to a time axis and defined as an observation signal sequence. (**c**) The intensity of a reconstruction image is determined by calculating the time correlation between a system function and an observation signal sequence.

In consideration of the abovementioned problems, we have proposed an image reconstruction method based on the correlation information between an observed signal (induced electromotive force: induced EMF) and a system function without depending on inverse matrix operations [12]. The conceptual diagram of this technique is shown in Figure 1. Here, for simplification, the analyzed matrix is assumed to include three points.

First, a system function is defined. When an MNP is arranged as a delta function at the left end matrix point (*i* = 1), a field free point (FFP) [6,7] where the local magnetic field strength is almost zero is scanned in order (*x* = 1, 2, 3) while applying an alternative magnetic field at each FFP. Here, although such a procedure may be classified under the category of narrow band MPI [20], the FFP scanned by our method is encoded intermittently as in robot position movement [7]. Consequently, a series (*G*_*i=1*_) that combines three waveforms of the induced EMF observed at each FFP is created. The system function at each matrix point (*i* = 2, 3) is defined in a similar manner, with the position of an MNP being changed and the series *G*_*i=2*_ and *G*_*i=3*_ being created, respectively (Figure 1(a)). Next, the induced EMF generated from the unknown MNPs’ distribution is observed at each FFP (*x* = 1, 2, 3), and it is considered as the series *V* connected to the time-axis as well as the abovementioned system function. Here, the observed signal *V* shown in Figure 1(b) reflects the outline form of the signal series obtained when the MNP is arranged at the left end matrix as an example. Then, the correlation information of this observed signal and each system function is calculated (Figure 1(c)). It is expected that only the magnetization signal generated from a target region is emphasized and reflected as the image intensity by such correlation processing. In contrast, an interference signal is difficult to reflect as the reconstructed image intensity because the correlation between the observed waveform of the induced EMF and the system function is small. In the case of a general two-dimensional image, the image intensity *F*(*i*, *j*) in the proposed method can be expressed by the following equation:

(1) $$F\left(i,j\right)=\int V_{x,z}\left(t\right)G_{i,j;x,z}\left(t\right)\mathrm{dt}$$

Here, *x* and *z* express the scanning position of FFP, *V*_*x,z*_ expresses an observed signal, and *G*_*x,z*_ expresses the system function as follows [12].

(2) $$G_{i,j;x,z}\left(t\right)=G_{i,j}\left[\mathrm{xt}+X\left(z-1\right)\right],\;\begin{array}{c} x=1,2,\cdots ,X\\ z=1,2,\cdots ,Z \end{array}\;$$

(3) $$V_{x,z}\left(t\right)\equiv V\left[\mathrm{xt}+X\left(z-1\right)\right],\;\begin{array}{c} x=1,2,\cdots ,X\\ z=1,2,\cdots ,Z \end{array}$$

## Methods

### Evaluation of magnetizing properties of MNP

**Figure 2:**
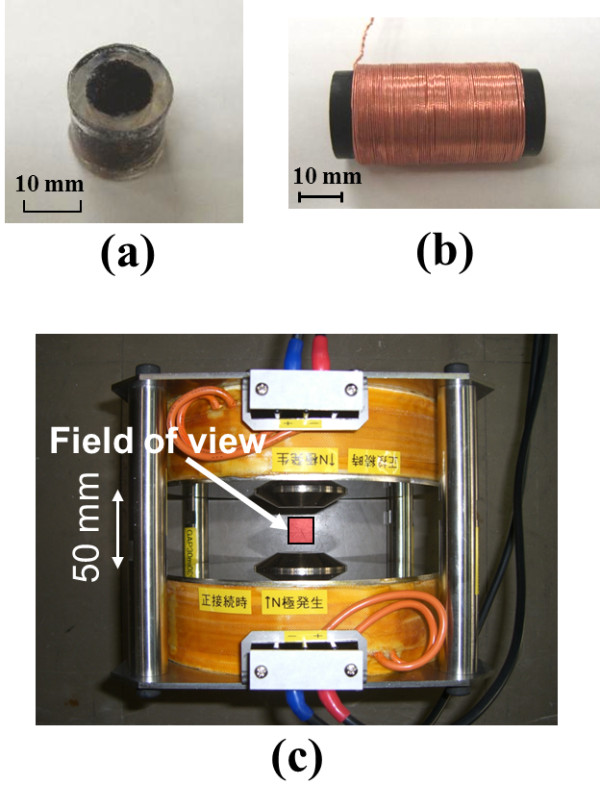
**Outside view of phantom and MPI prototype system.** (**a**) Each MNP sample was placed in an acrylic cylindrical container. (**b**) The receiver coil was coaxially arranged on a cylindrical container placed in the MNP sample. (**c**) The prototype system was built to collect one-dimensional MPI data.

In this study, four types of samples (including ferucarbotran), as listed in Table 1, with ferucarbotran as the base material and adjusted particle diameters were used. These samples were respectively prepared by magnetic separation, centrifugal separation, and gel filtration. The Fe concentration of each sample as well as the Resovist sample was adjusted to 28 [mg/mL]. The average diameter including the coating layer (Da) and the particle size distribution of the average diameter including the coating layer (Dv) were evaluated using a photon correlation spectrometer, the susceptibility was measured using the magnetic balance method, and the *T*_*2*_ relaxation time was evaluated using 0.47 [T] NMR equipment. The polydispersity index (PI) was evaluated using the light scattering method. A vibrating sample magnetometer (VSM) is commonly used for evaluating the magnetization properties; however, in this case, these properties were evaluated using our MPI prototype because the detection sensitivity in MPI was also evaluated. Each sample was sealed hermetically in 0.7 [cc] cylindrical containers (∅5 [mm], approximately 12 [mm] in length) made from acrylics, and a solenoid coil of 19 [mm] diameter and with 350 turns was arranged as a receiver coil on the outer circumference (Figure 2). They were installed centered on the gap (50 [mm]) of a customized Maxwell pair coil (Toyojiki Industry Co., Ltd., Niiza, Japan) with an iron core, 180 [mm] diameter, and 285 turns for each coil. An alternative magnetic field with an amplitude of approximately 65 [mT] was generated at the center of those coils by applying an alternative current with an amplitude of 12.0 [A] and frequency of 33.0 [Hz] to each coil in the same direction.

**Table 1:** Characteristics of each sample based on ferucarbotran

**No.**	**D**_**a **_**[nm]**	**PI**	**D**_**v **_**[nm]**	**Magnetic susceptibility [erg・gauss**^**-2**^**・g**^**-1**^**]**	**T**_**2 **_**relaxivity [mM**^**-1**^**s**^**-1**^**]**	**D [nm]**
1	55	0.28	30-110	0.0315	186	15
2	59	0.24	30-120	0.0387	268	18
3	86	0.19	50-200	0.0399	494	20
4	56	0.26	35-110	0.0354	274	17

*D*_*a*_ Average diameter including coating layer.

*D*_*v*_ Particle size distribution of average diameter including coating layer.

*D* Particle diameter of experimentally evaluated MNP.

To distinguish between the magnetization components (harmonics) generated from an MNP and the primary magnetic field components applied from the outside, the induced EMF to a coil without a sample was observed previously, and it was defined as the raw flux density applied to an MNP. Then, an induced EMF was generated when an MNP was arranged, and the actual induced EMF generated from the MNP was determined from the difference between this observed signal and the above-described raw flux density. In this case, the absolute value of flux density was corrected using a gauss meter (Model 460; Lakeshore Cryotronics Inc., OH, USA).

The average particle diameter of each sample was computed by approximating the magnetization curve obtained in the abovementioned experiment with a Langevin function.

### Evaluation of image reconstruction method by numerical analysis

A gradient magnetic field intensity of 1.5 [T/m] at the center of a Maxwell pair coil and an alternating magnetic field intensity of 32.0 [mT] were used. The FOV was set as 40 [mm] × 40 [mm], and the matrix size was set as 21 × 21. The system function was analytically computed in each matrix point of this FOV based on the Langevin function approximated using the particle diameter of each MNP as evaluated by the abovementioned procedure.

Then, based on equations (1), (2) and (3) and Figure 1, image reconstruction was performed for the signal series that connected the induced EMF observed at the FFP scanned by each matrix point.

### Evaluation of one-dimensional reconstructed image using prototype

Both an alternative magnetic field and an FFP were generated using the abovementioned one-axis Maxwell pair coil. The coil current was supplied from a bipolar power supply (BP30-30; Heiwa Electric Co., Ltd., Kashiwa, Japan) in constant current mode, and the current wave for scanning the FFP was controlled by a function generator (AFG3252; Tektronix, Inc., OR, USA). After the induced EMF was detected using the receiver coil (diameter 19 [mm], 350 turns) and passed through the programmable filter (3628, NF Corporation, Yokohama, Japan), it was supplied to a 14-bit AD converter (M2i4031; Spectrum Systementwicklung Microelectronic GmbH, Grosshansdorf, Germany). The detected signal was sampled with a sampling frequency of 20 [kHz] and was sent to a personal computer (dc7800 MT/CT; Hewlett–Packard Co., CA, USA).

As a result of arranging each abovementioned sample at the center of the gap of a Maxwell pair coil and applying the alternative current with an amplitude of 6.0 [A] and frequency of 39.0 [Hz] in the same direction, the MNPs in each sample were subjected to an alternative magnetic field of approximately 30 [mT]. Under such device conditions, a gradient magnetic field of 1.9 [T/m] was generated by applying an offset current of 12.0 [A] simultaneously to the opposite direction of each coil, and an FFP was formed at the center of the Maxwell pair coil.

In a preliminary experiment, the electric current required to move the FFP by a unit length (this corresponds to the spatial resolution) was evaluated; based on this, the scanning of the FFP was controlled by the function generator. In addition, a ±20-mm region from the Maxwell pair coil’s center was set as the FOV, and the matrix that divides the inside of this FOV into 21 points was made into each measuring point (FFP).

To detect only the induced EMF generated from an MNP in consideration of the frequency purity of the alternative magnetic field due to the imperfection of the power supply and the coil, difference processing with the induced EMF and without a sample was carried out.

## Results and discussion

### Magnetization property of each MNP sample

**Figure 3:**
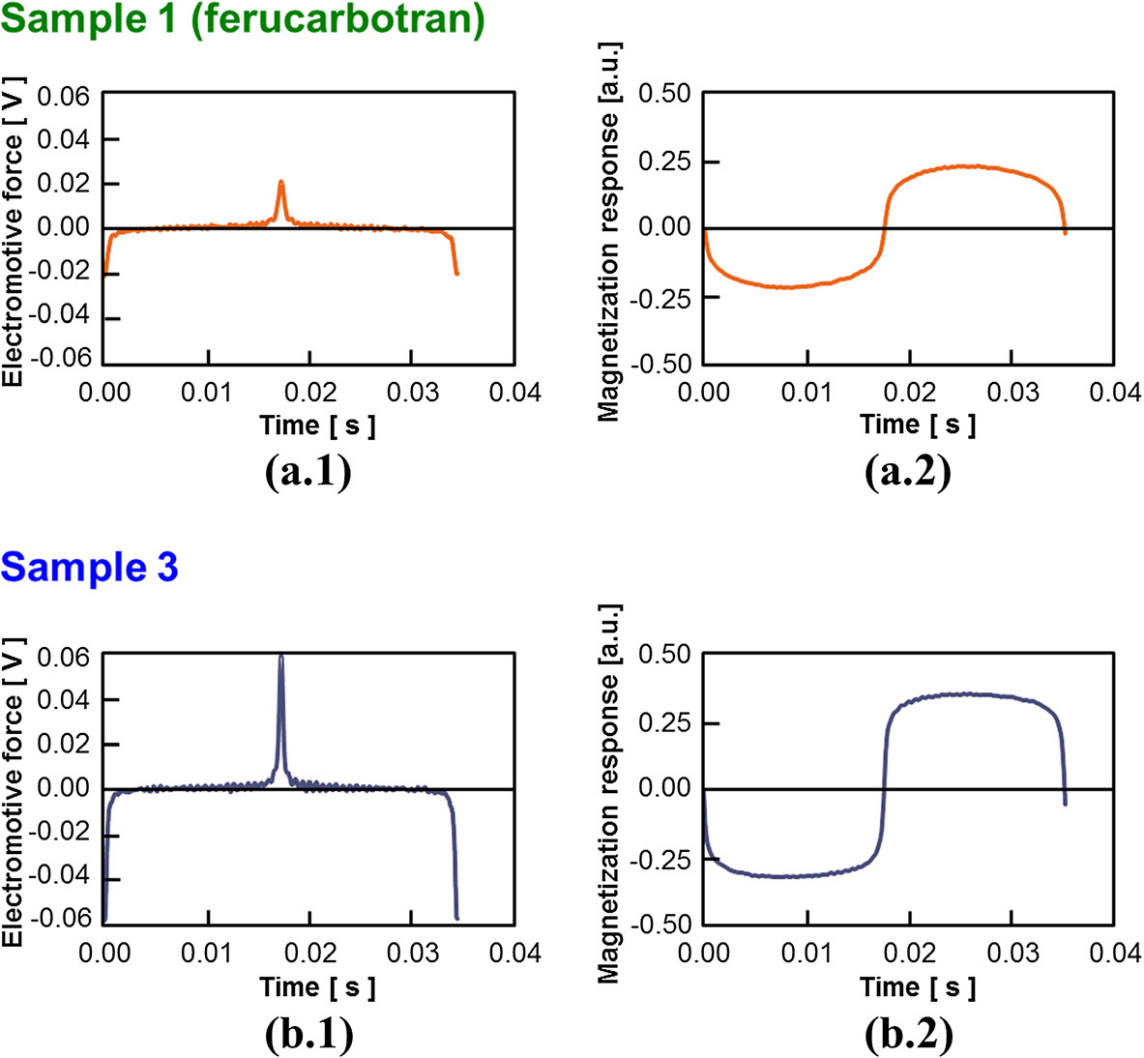
**Signal detected with each sample. (a.1**), (**b.1**) Waveform of induced electromotive force. (**a.2**), (**b.2**) Magnetization response waveform.

**Figure 4:**
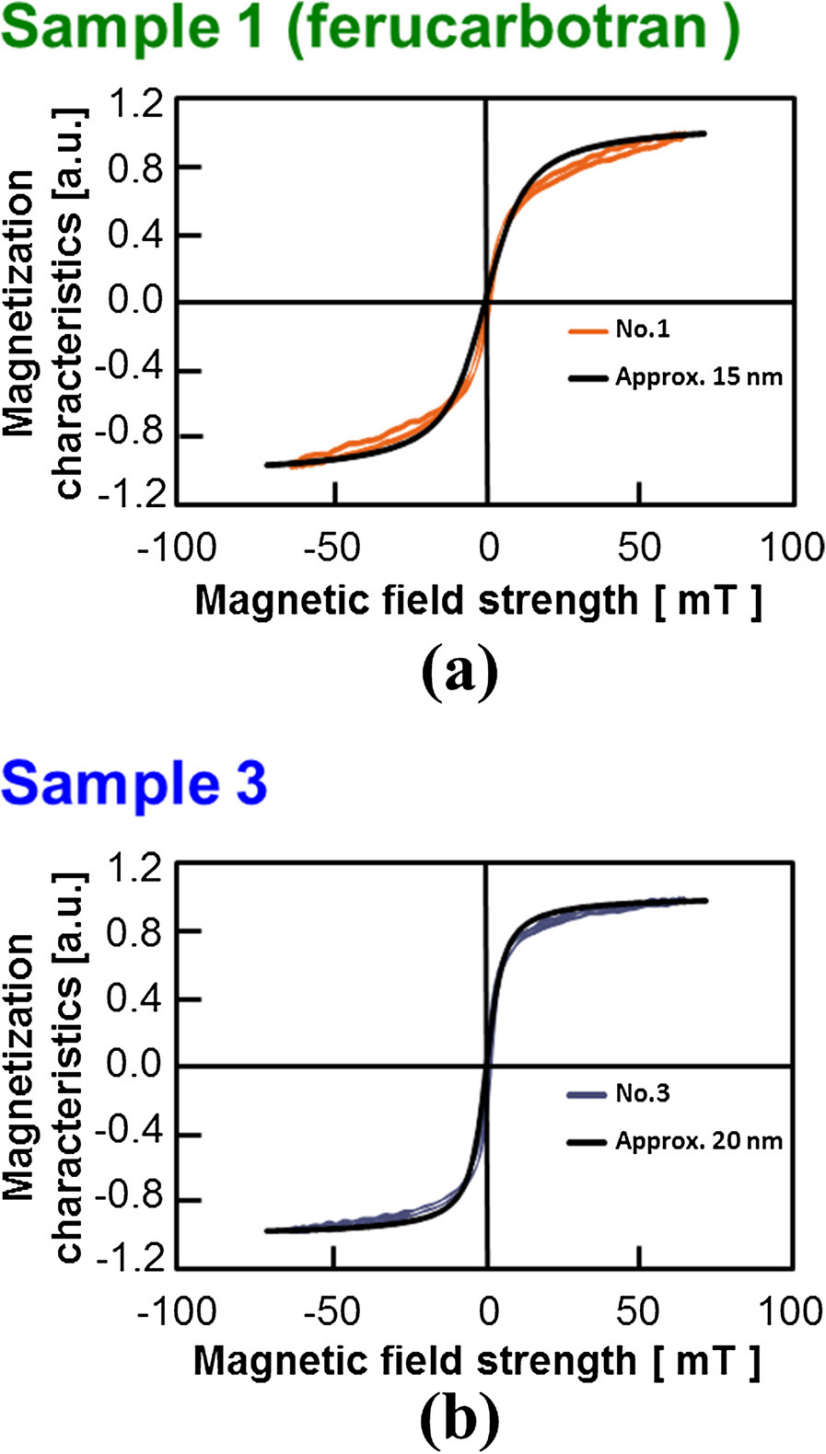
Magnetization curve of each sample.

The induced EMF from the samples made with ferucarbotran as the base material to the external alternative magnetic field and the magnetization response obtained from the integration operation of EMF are shown in Figure 3 (in what follows, only the results of sample 1 (ferucarbotran) and sample 3, which show the characteristic tendency, were displayed.). In addition, the average particle diameter of each sample was evaluated by comparing the observed magnetization properties with the magnetization curve of the MNP as indicated by Langevin’s approximate expression (Figure 4). These magnetization curves were normalized by the maximum magnetization of each case. The results are summarized in Table 1.

It was estimated that the average particle diameter *D* of sample 1 (ferucarbotran), which has been clinically approved, was 15 [nm]. That of sample 3 was 20 [nm], which is the largest among all samples, and it was shown that sample 3 is the most suitable because a sample with large particle diameter is advantageous for MPI. Here, the difference between the obtained magnetization properties and the approximated curve reflected the variation in the particle size distribution of the MNPs in a sample, and it was suggested that the particle diameter of sample 3 was adjusted satisfactorily. This result was also supported by the evaluation results obtained by PI and *D*_*v*_, shown in Table 1. Because the particle size distribution follows a logarithmic normal distribution [21], it was considered important to adjust the particle diameter in a sample uniformly in order to improve the image resolution in MPI. Moreover, the induced EMF detected from sample 3 was approximately 3 times that from ferucarbotran, indicating that sample 3 also contributes greatly to the improvement of SNR. This is based on the increase in the saturation magnetization of the MNP accompanying the increase in the particle diameter [15].

From the physical properties obtained by this evaluation, as listed in Table 1, it was confirmed that the particle diameter of MNPs was also related to the susceptibility and *T*_*2*_ relaxation time.

### Numerical analysis of time-correlation MPI image reconstruction

**Figure 5:**
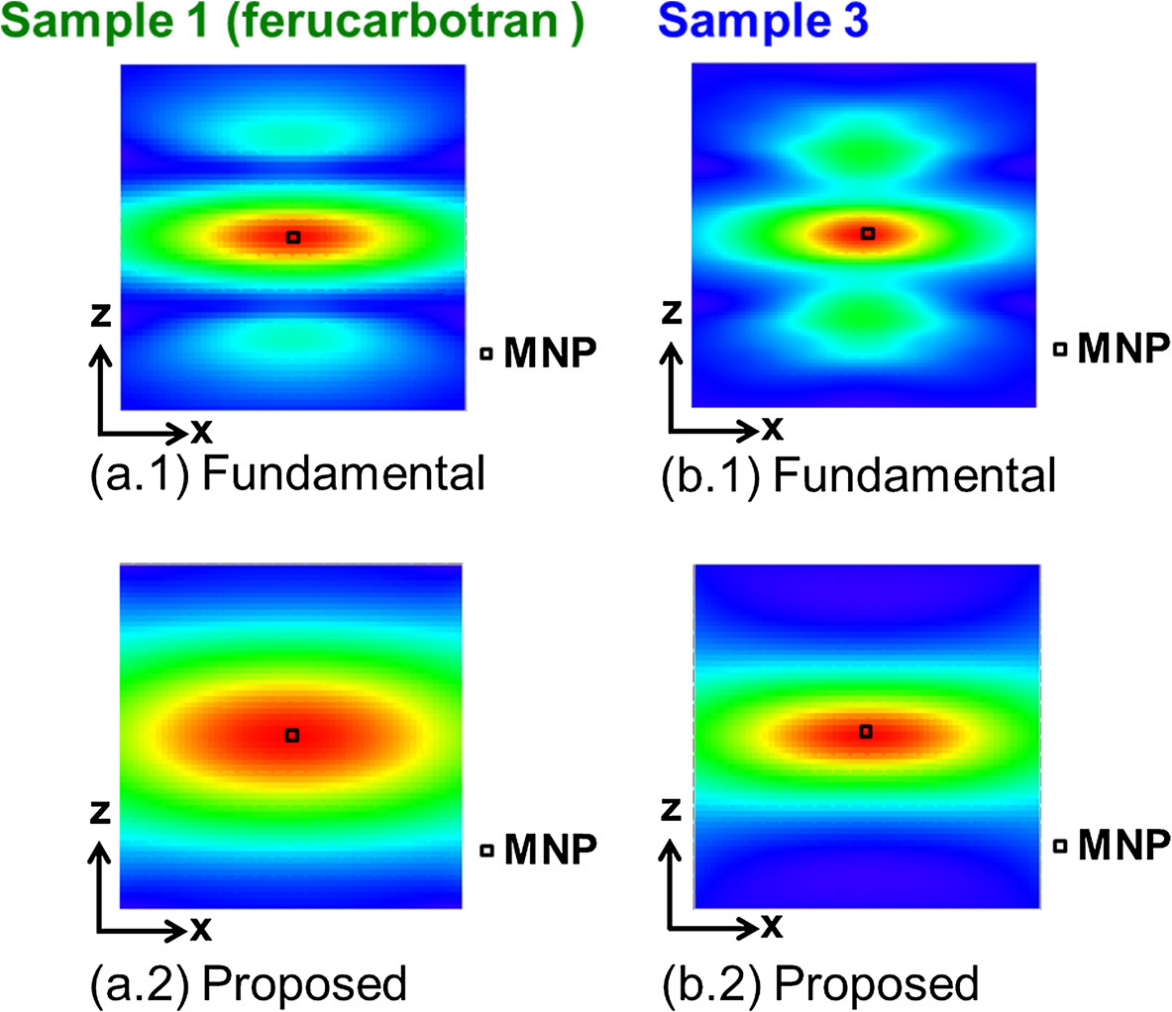
**Reconstructed image for each sample by numerical analysis.** (**a.1**), (**a.2**) Images by fundamental and proposed reconstruction methods in the case of sample 1, respectively. (**b.1**), (**b.2**) Images by fundamental and proposed reconstruction methods in the case of sample 3, respectively.

**Figure 6:**
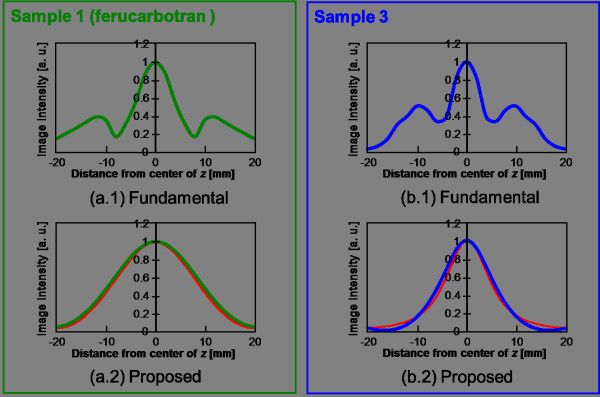
**Image profiles of reconstructed images by numerical analysis.** (**a.1**), (**a.2**) Image profiles at *z* = 0 in the case of sample 1, respectively. (**b.1**), (**b.2**) Image profiles at *z* = 0 in the case of sample 3, respectively. (**a. 2**), (**b. 2**) Individual theoretical point spread functions indicated by red lines.

Next, the image reconstruction results of the numerical simulation based on the characteristics of sample 1 (ferucarbotran) and sample 3, which were chosen from among all samples as discussed above, are described. The images reconstructed by the fundamental image reconstruction method based on an imaging principle (an alternative magnetic field was applied at each FFP that was scanned for every encoded position [7]) and our proposed time-correlation method for a sample arranged at the center pixel of the FOV are shown in Figure 5. In addition, the profiles at *z* = 0 of these reconstruction images are shown in Figure 6. It was confirmed that the image artifact observed in the upper and lower sides of the actual MNP by the fundamental image reconstruction method was suppressed by the proposed method. However, the image resolution of the proposed method (~12 mm) was slightly degraded compared to that of the fundamental method (~8 mm) using the FWHM for sample 1. Then, it was shown that the image blurring increased. This is because theoretically, the distribution of correlation between the observed signal and the system function at every FFP position (Figure 1) was given as the image intensity with nearly two times the FWHM of the system function [22]. The PSF of this image reconstruction method was expected as an autocorrelation distribution of the system function, and it was superimposed in Figure 6 for MNPs of each particle diameter. Therefore, it is possible to newly propose the following iterative estimation of the distribution of MNPs in order to improve the image resolution [13].

(1)The position at which the correlation with an observed signal and a system function is the maximum in the FOV is detected.

(2)At this position (image matrix), the amount of correlation is given as the reconstructed image intensity.

(3)The distribution of a corresponding system function is subtracted from the observed signal at this position.

Then, the candidate of the processing position is moved to the next FFP and the abovementioned process is repeated until the residual of the subtraction signal at every FFP becomes small. It is considered that a deconvolution with a system function can be carried out equivalently by such processing; therefore, the image blurring can be reduced effectively without inverse matrix operations.

**Figure 7:**
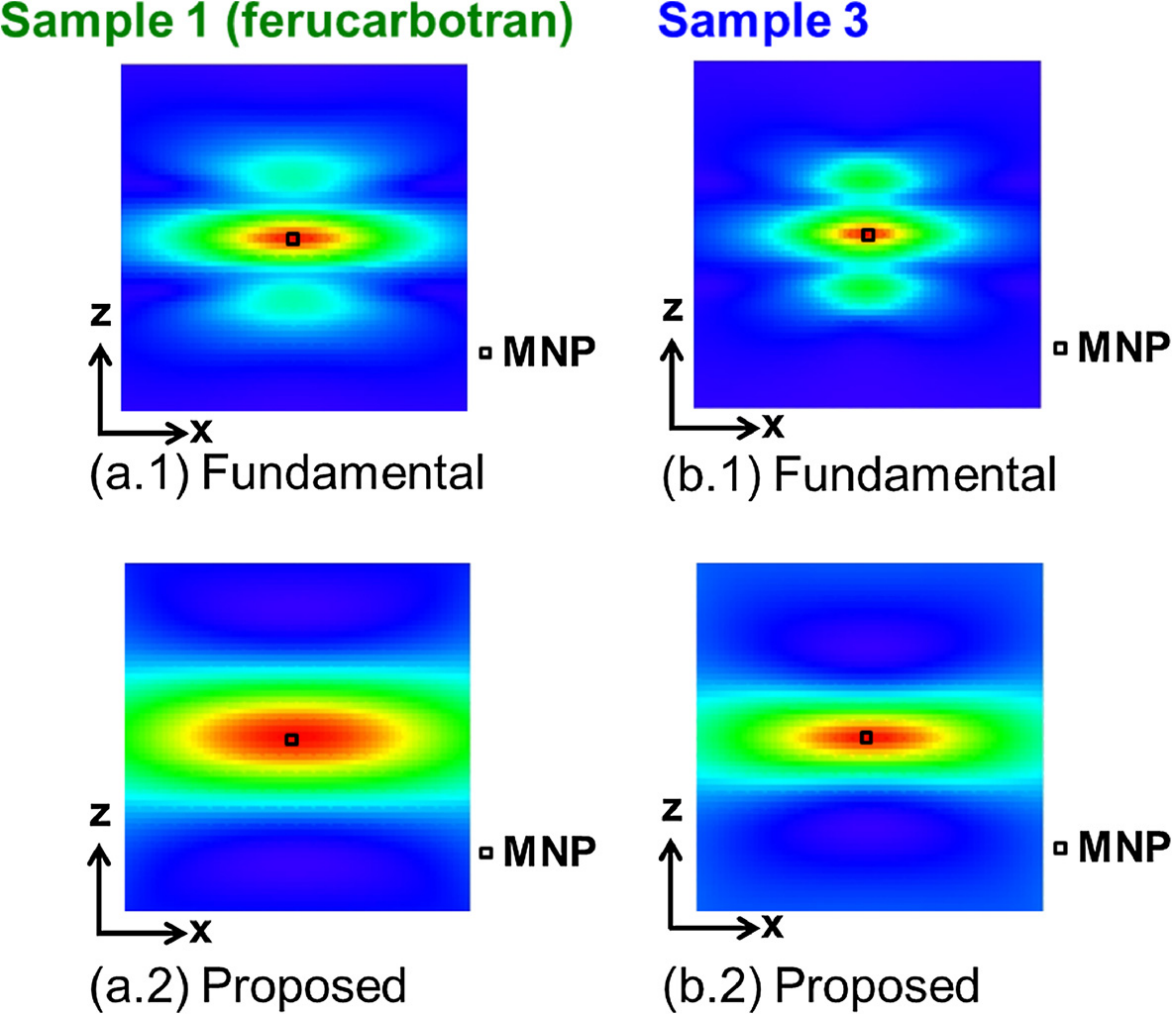
**Reconstructed image with numerical analysis for each sample at gradient field of 2.5 T/m.** (**a.1**), (**a.2**) Images by fundamental and proposed reconstruction methods in the case of sample 1, respectively. (**b.1**), (**b.2**) Images by fundamental and proposed reconstruction methods in the case of sample 3, respectively.

An evaluation of the profile at *z* = 0 (Figure 6) showed that the spatial resolution of sample 3 was approximately 1.3 times better than that of sample 1. This corresponded to the fact that the spatial resolution obtained by a gradient magnetic field strength of 2.5 [T/m] for sample 1 was achieved by a gradient magnetic field strength of approximately 1.5 [T/m] for sample 3 (Figure 7). In other words, it was shown that the dependence on hardware requirements was reduced by 0.6 times under such conditions and that the spatial resolution could be improved by simply adjusting the particle diameter.

### One-dimensional imaging experiment using prototype

**Figure 8:**
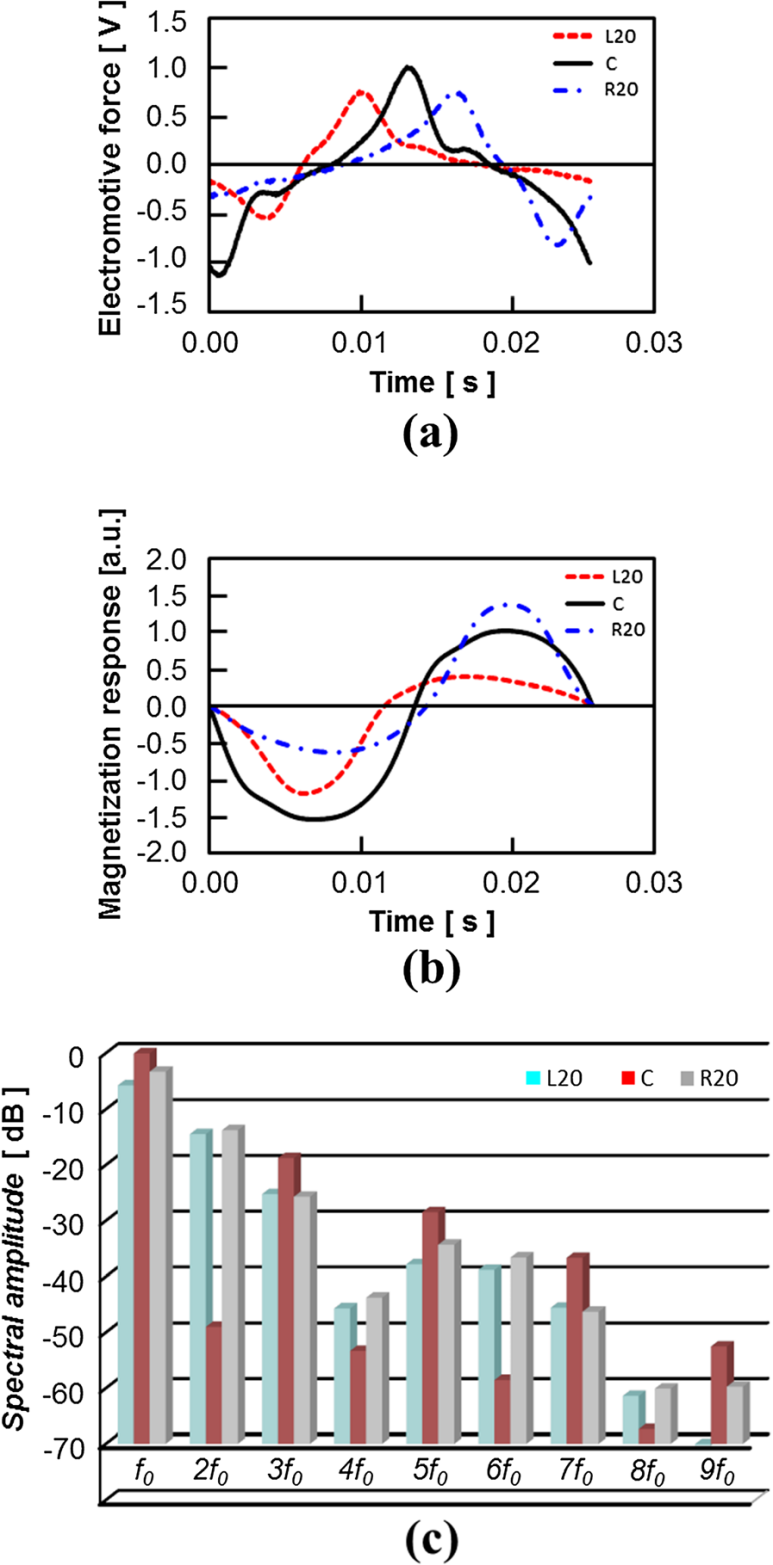
**Signal detected using prototype.** (**a**) Waveform of induced electromotive force. (**b**) Magnetization response waveform. (**c**) Fourier components of magnetization response waveform. Here, only the waveforms obtained by scanning the FFP at −20 [mm] (L20), 0 [mm] (C), and 20 [mm] (R20) along the *z*-axis were indicated.

**Figure 9:**
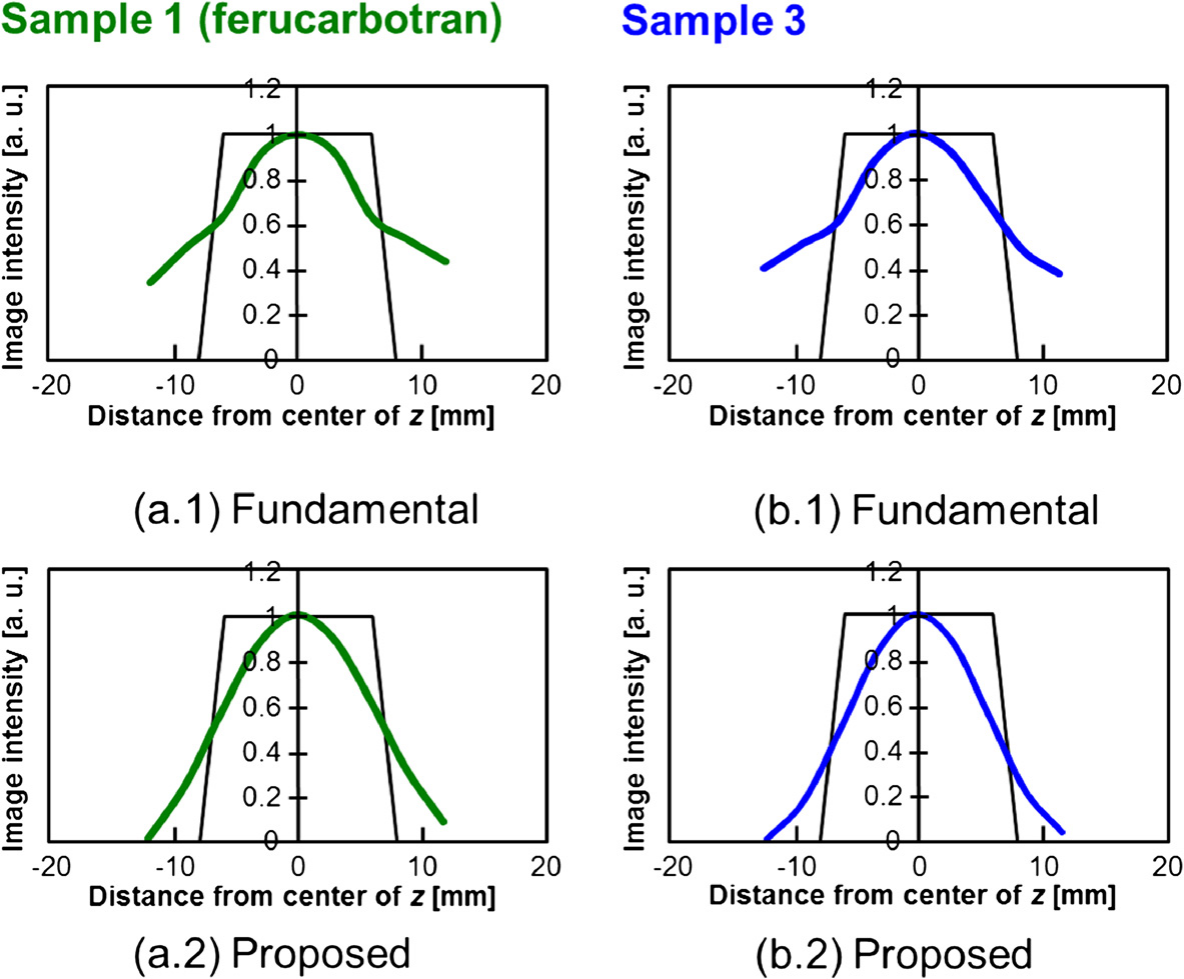
**One-dimensional reconstructed images using prototype.** (**a.1**), (**a.2**) Images by fundamental and proposed reconstruction methods in the case of sample 1, respectively. (**b.1**), (**b.2**) Images by fundamental and proposed reconstruction methods in the case of sample 3, respectively. In these cases, the calculated system function corresponding to each particle diameter was used.

The waveform of the induced EMF obtained by scanning the FFP, the magnetization response calculated from the integration of the EMF, and the Fourier transform of this magnetization response are shown in Figure 8. Here, when the center coordinate of the FOV was set to 0, only the wave obtained by scanning the FFP at −20 [mm], 0 [mm], and 20 [mm] along the *z*-axis was indicated in the figure (this corresponds to the sign L20, C, and R20, respectively.). It was confirmed that the difference of the induced EMF’s waveform in an experiment appeared depending on the position of the FFP. In other words, because it would reflect that the position of the MNP was indistinguishable when the correlation with an observed signal was evaluated with a system function, the validity of our proposed method based on time-correlation information was also confirmed by experimental data. With regard to sample 1 (ferucarbotran) and sample 3, the one-dimensional images obtained using the fundamental image reconstruction method and the proposed method are shown in Figure 9. In these reconstructed images, although the precise system function should ideally be measured from the signal observed when the MNPs are arranged in a shape like a delta function at each analyzed point in the reconstructed image, the analytically calculated system function corresponding to each particle diameter at every image matrix point was used. This is because considerable time is generally required for the measurement of a system function, and our system’s operation might become unstable over such a long time. It was found that sample 3 afforded an image resolution superior to that of sample 1 through both the experimental results as well as the numerical simulation.

## Conclusions

In this study, to suppress image artifacts in MPI and improve the spatial resolution without requiring high-performance hardware, an image obtained using an MNP sample made of biocompatible ferucarbotran as the base material and a sample made by our proposed method were compared. It is essential to evaluate the biocompatibility because a large-diameter nanoparticle may behave differently in internal organs compared to Resovist, which is approved for clinical use. Nonetheless, some samples used for MPI were found to have magnetizing properties superior to those of ferucarbotran. Consequently, it was shown that an equivalent image quality (spatial resolution) could be obtained with a smaller gradient magnetic field strength compared to the case in which ferucarbotran was used.

Therefore, if a method for preparing MNP samples consisting of particles with large and equisized diameters made from ferucarbotran as the base material is developed, the MPI image quality can be further improved, and the conditions imposed on hardware ability can be reduced. However, because our study was restricted to the use of an alternating magnetic field with relatively low frequency, evaluations in consideration of the dynamic characteristic of the MNP [19,23] will be required when faster scanning of FFP is used. In addition, because a sufficiently high spatial resolution has not yet been achieved theoretically using the proposed time-correlation MPI reconstruction method, it is necessary to devise an algorithm that can use correlation information more effectively.

## Competing interests

The authors have no competing interests to declare.

## Authors’ contributions

YI conceived the study, built the algorithm, and drafted the manuscript. TH implemented the algorithm in a program, developed the prototype system, performed data collection, and helped to draft the manuscript. SN prepared the experimental MNP samples and helped to draft the manuscript. All authors have read and approved the final manuscript.

## Pre-publication history

The pre-publication history for this paper can be accessed here:

http://www.biomedcentral.com/1471-2342/13/15/prepub

## References

[B1] Huang X Jain PK El-Sayed H El-Sayed MA Gold nanoparticles: interesting optical properties and recent applications in cancer diagnostics and therapy Nanomedicine 2007 2 681 693 10.2217/17435889.2.5.681 17976030

[B2] Dougherty TJ Gomer CJ Henderson BW Jori G Kessel D Korbelik M Moan J Peng Q Photodynamic therapy J Natl Cancer Inst 1998 90 889 905 10.1093/jnci/90.12.889 9637138 PMC4592754

[B3] El-Sayed IH Huang X El-Sayed MA Selective laser photo-thermal therapy of epithelial carcinoma using anti-EGFR antibody conjugated gold nanoparticles Cancer Lett 2006 239 129 135 10.1016/j.canlet.2005.07.035 16198049

[B4] Hergt R Hiergeist R Zeisberger M Schüler D Heyen U Hilger I Kaiser WA Magnetic properties of bacterial magnetosomes as potential diagnostic and therapeutic tools Journal of Magnetism and Magnetic Materials 2005 293 80 86 10.1016/j.jmmm.2005.01.047

[B5] Jordan A Scholz R Maier-Hauff K Johannsen M Wust P Nadobny J Schirra H Schmidt H Deger S Loening S Lanksch W Felix R Presentation of a new magnetic field therapy system for the treatment of human solid tumors with magnetic fluid hyperthermia Journal of Magnetism and Magnetic Materials 2001 225 118 126 10.1016/S0304-8853(00)01239-7

[B6] Gleich B Method for determining the spatial distribution of magnetic particles DE 10151778(A1)05 2003 05 08

[B7] Gleich B Weizenecker J Tomographic imaging using the nonlinear response of magnetic nanoparticles Nature 2005 435 1214 1217 10.1038/nature03808 15988521

[B8] Weizenecker J Gleich B Rahmer J Dahnke H Borgert J Three-dimensional real-time *in vivo* magnetic particle imaging Phys Med Biol 2009 54 L1 L10 10.1088/0031-9155/54/5/L01 19204385

[B9] Schmale I Rahmer J Gleich B Kanzenbach J Schmidt JD Bontus C Woywode O Borgert J First phantom and in vivo MPI images with an extended field of view 2011 Florida: Proc. of SPIE Medical Imaging 796510.6

[B10] Kusayama Y Ishihara Y A preliminary study on the molecular imaging device using magnetic nanoparticles. *Technical Report of IEICE* MBE 2007 107 15 18

[B11] Kusayama Y Ishihara Y High-resolution image reconstruction method on the molecular imaging using magnetic nanoparticles IEICE Trans. D 2009 J92–D 1653 1662

[B12] Ishihara Y Kuwabara T Honma T Nakagawa Y Correlation-based image reconstruction methods for magnetic particle imaging IEICE Transactions on Information and Systems 2012 E95-D 872 879 10.1587/transinf.E95.D.872

[B13] Honma T Shimizu S Ishihara Y Kyoso M Correlation-based image reconstruction methods for magnetic particle imaging Proceedings of the Branch Conference of Japanese Society for Medical Biological Engineering 2012 2012 Tokyo 2012: C-1-04

[B14] Goodwill PW Conolly SM The X-space formulation of the magnetic particle imaging process: 1-D signal, resolution, bandwidth, SNR, SAR, and magnetostimulation IEEE Trans Med Imaging 2010 29 1851 1859 20529726 10.1109/TMI.2010.2052284

[B15] Chikazumi S Charap SH Physics of Magnetism 1964 New York: John Wiley and Sons

[B16] Ferguson RM Minard KR Krishnan KM Optimization of nanoparticle core size for magnetic particle imaging J. Magn. Magn. Mater 2009 321 1548 1551 10.1016/j.jmmm.2009.02.083 19606261 PMC2709850

[B17] Ferguson RM Minard KR Khandhar AP Krishnan KM Optimizing magnetite nanoparticles for mass sensitivity in magnetic particle imaging Med Phys 2011 38 1619 1626 10.1118/1.3554646 21520874 PMC3064684

[B18] Eberbeck D Wiekhorst F Wagner S Trahms L How the size distribution of magnetic nanoparticles determines their magnetic particle imaging performance Appl Phys Lett 2011 98 182502.1 182502.3

[B19] Ludwig F Wawrzik T Yoshida T Gehrke N Briel A Eberbeck D Schilling M Optimization of magnetic nanoparticles for magnetic particle imaging IEEE Transactions on Magnetics 2012 48 3780 3783

[B20] Goodwill PW Scott GC Stang PP Conolly SM Narrowband magnetic particle imaging IEEE Trans Med Imaging 2009 28 1231 1237 19211340 10.1109/TMI.2009.2013849

[B21] Chantrell RW Popplewell J Charles SW Measurements of particle size distribution parameters in ferrofluids IEEE Transactions on Magnetics 1978 14 975 977 10.1109/TMAG.1978.1059918

[B22] Yarlagadda RKR Analog and Digital Signals and Systems 2010 New York: Springer Science + Business Media

[B23] Yoshida T Enpuku K Ludwig F Dieckhoff J Wawrzik T Lak A Schilling M Characterization of Resovist® nanoparticles for magnetic particle imaging Springer Proceedings in Physics 2012 140 3 7 10.1007/978-3-642-24133-8_1

